# Fluorescence study of freeze-drying as a method for support the interactions between hyaluronan and hydrophobic species

**DOI:** 10.1371/journal.pone.0184558

**Published:** 2017-09-08

**Authors:** Petra Michalicová, Filip Mravec, Miloslav Pekař

**Affiliations:** Brno University of Technology, Faculty of Chemistry, Institute of Physical and Applied Chemistry and Materials Research Centre, Brno, Czech Republic; Brandeis University, UNITED STATES

## Abstract

A freeze-drying method enabling solubilization of hydrophobic species in aqueous solutions of native hyaluronan is described. The method is based on opening the access to supposed hydrophobic patches on hyaluronan by disturbing its massive hydration shell. Hydrophobic and/or polarity-sensitive fluorescence probes were used as hydrophobic models or indicators of interactions with hydrophobic patches. Fluorescence parameters specific to individual probes confirmed the efficiency of the freeze-drying method. This work is the first step in developing biocompatible and biodegradable carriers for hydrophobic drugs with targeted distribution of the active compound from native, chemically non-modified hyaluronan.

## Introduction

**Fig 1:**
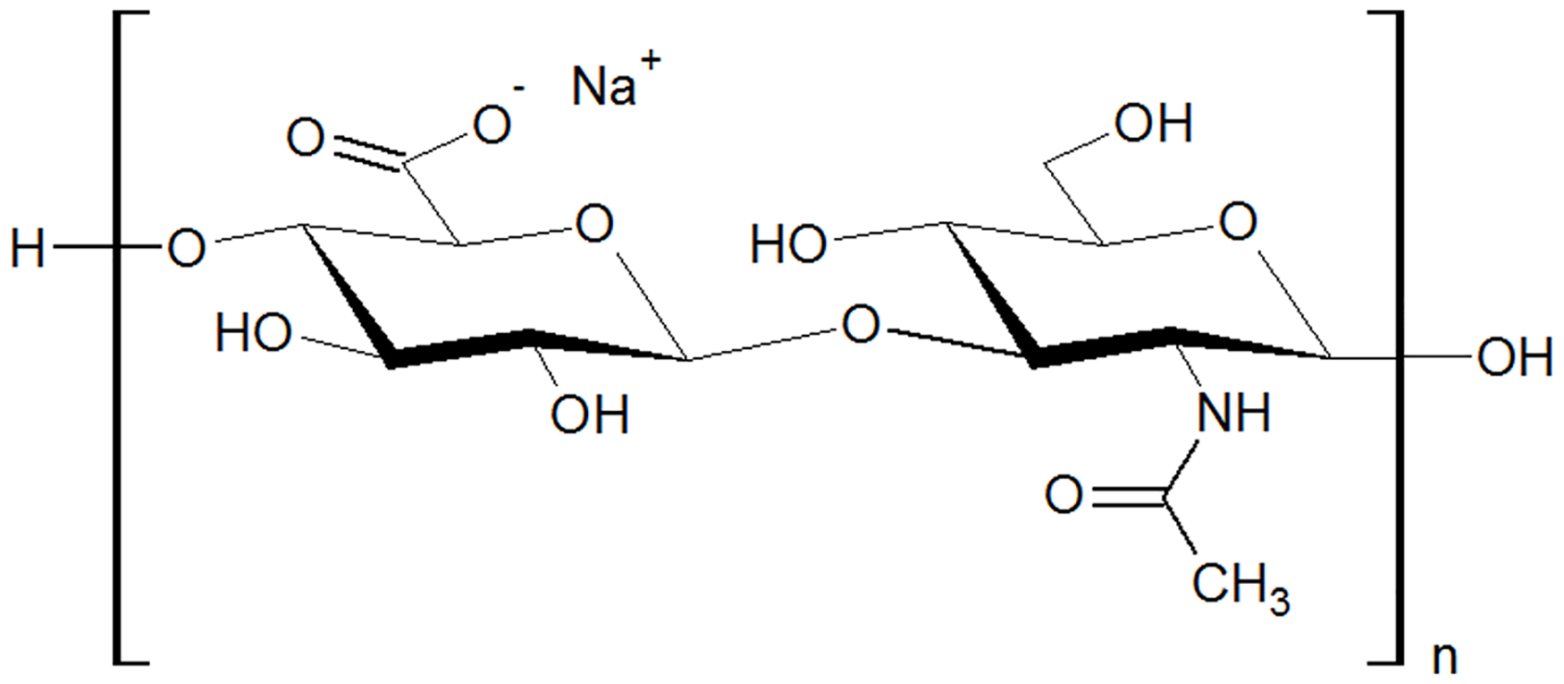
Structure of hyaluronan biopolymer.

Hyaluronan is a biopolymer which can be characterized as essential for living organisms of various complexity, ranging from microorganisms to vertebrates. It is a linear biopolysaccharide composed of D-glucuronic acid and N-acetyl-D-glucosamine linked by a β-1,3 glycosidic bond (Fig 1). These disaccharide units are linked by β-1,4 bonds. It is a part of the extracellular matrix in most tissues and also a major component of a variety of other tissues. Besides its mechanical functions, this compound is important for many biological processes [1–3]. Its contribution to the proliferation of tumour cells is one typical example. This area of cancer research involving hyaluronan is the subject of many studies. Literature shows that cancerous tissues are rich in the receptors CD44 and RHAMM [4–7]. These receptors are specific to hyaluronan and cause the packaging of tumour tissue using the biopolymer. This is the main reason for the application of hyaluronan in the area of drug-delivery systems (DDS) [8–12]. The function of DDS can be compared to a Trojan horse—tumour cells interact with hyaluronan from the surrounding cellular environment through the abovementioned receptors. Systems containing hyaluronan and cytostatic drugs release the drug and subsequently induce cell death. The majority of already utilized or potential drugs have a hydrophobic character. In contrast, hyaluronan is a highly hydrophilic polymer. Two strategies are used to tackle this problem—to prepare a hyaluronan-drug conjugate or to hydrophobize hyaluronan [13]. In the latter, hydrophobically modified hyaluronan can form polymeric micelles in an aqueous medium capable of solubilizing hydrophobic substances [14–17]. Both approaches are based on chemical reactions of hyaluronan conducted usually in some organic solvent. Chemical modification, however, can affect the biological functions and biocompatibility of hyaluronan; further, the organic solvent should be carefully removed from a product intended for use in the human body [13]. An alternative is to use hyaluronan-surfactant complexes formed by physical (electrostatic and hydrophobic) interactions [18–20]. However, due to hyaluronan’s negative charge, cationic surfactants should be used which have general cytotoxic effects though somewhat moderated in the presence of hyaluronan [21].

Scott published an idea about existence of “hydrophobic patches” on the hyaluronan chain [22]. Because of the beta configuration, all bulky groups occupy sterically favorable equatorial positions. On the other hand, small hydrogen atoms are moved into less sterically favorable axial positions. This gives rise to the amphiphilic character of the hyaluronan chain, which contains both a hydrophilic (prevalent) part and a hydrophobic part. We therefore hypothesized that these hydrophobic patches could be used to bind non-polar substances directly onto the native hyaluronan chain. However, in aqueous solution, the chain generates a twisting ribbon structure in which the hydrophilic face is oriented into the solution and the hydrophobic face is hidden inside the domain. The non-polar areas are then probably protected also by hydration shell—hyaluronan is reported to have a thick hydration layer resulting in a large hydrodynamic volume [23–26]. Therefore, interactions between native hyaluronan and hydrophobic species must somehow be supported by opening the hydration shell in order to make the hydrophobic patches accessible for interactions. The combination of hydrophilic and hydrophobic character as an intrinsic property of the hyaluronan backbone was demonstrated by atomic force microscopy investigations of hyaluronan deposited on various surfaces [27, 28]. The behavior of graphite deposition was very different from that on mica. Hyaluronan deposited on hydrophobic graphite surface without rinsing with water before drying interacted more strongly with the surface than the well-rinsed depositions. The authors hypothesized that hyaluronan chains in (excess) water tended to mask the hydrophobic patches and their interactions with the hydrophobic substrate were weakened.

In this work, a freeze-drying method in the presence of an organic co-solvent was studied as a potential opener of the hydration shell and supporter of hydrophobic interactions between the hyaluronan chain and non-polar species. Recently, Průšová et al. studied the effect of various drying techniques on the physical structure of native hyaluronan in solid state [29]. They demonstrated that the supramolecular structure of native hyaluronan can be modified by the selection of drying conditions including the freeze-drying. The authors also suggested that drying can represent a potential strategy for application of native hyaluronan in various areas including the pharmaceutical industry. Some studies reported potential degradation of hyaluronan during freeze-drying but their results are contradictory. Wedlock reported about 70% decrease in molecular weight of sodium hyaluronan whereas Tokita found only a minor depolymerization (about 2%) for the sodium form in contrast to the protonated form for which a decrease of molecular weight in about 80% was determined [30, 31]. Nevertheless, we included study of the effect of molecular weight in this work; moreover, alcohols were shown to protect the depolymerization [30] and, as explained below, tert-butanol was a component of freeze-dried compositions.

Freeze-drying is a method widely used in the pharmaceutical and biotechnological industries. The main application is in the careful removal of solvents and in improving the stability of the product. Systems with viruses, vaccines, proteins, or colloidal particles are often freeze-dried during the process of developing their formulations. Freeze-drying is relatively slow and expensive; thus, it is used for products of particular importance with respect to their subsequent use, or for products sensitive to higher temperatures [32, 33]. In our case, the applied biopolymer—hyaluronan—undergoes degradation processes even at temperatures below 100°C. For the abovementioned reasons therefore, freeze-drying is a quite convenient way of drying hyaluronan. The process of freeze-drying runs at laboratory temperature and low pressure. Individual components are solubilized in a solvent and the sample is frozen. Subsequently, after the freezing is complete, the sample is heated at low pressure. During this process, the solvent is first sublimated and then desorbed. This yields a porous cake with a low moisture content. In these conditions, biological growth or chemical reactions are not supported. For this study, the most important feature of freeze-drying is the possibility of removing the shell of water molecules around the hyaluronan chain.

The entire procedure can be divided into three steps [34–36]–freezing, primary drying (the sublimation of the frozen solvent), and secondary drying (the desorption of absorbed water). The freezing step involves the cooling of the liquid solution and the formation of ice crystals. During the process of crystal growth, the concentration of the suspension and also its viscosity increases. Finally the mixture is solidified in entire volume. The character of the phase could be amorphous, crystalline, or a combination of both. At this stage, unfrozen absorbed water molecules still remain in the sample. The literature indicates that the amount of absorbed water is about 10–35%. Process of freezing can be represented by phase diagrams of the studied systems.

During the second step, the frozen samples are heated up to room temperature, still at low pressure, and consequently the solvent is sublimated. This means that the mass of the solvent is transferred directly from the solid phase to the gas phase. The sample must be held at low pressure to prevent it from melting. The released vapor is transferred through the dried product to the surface and continues into the instrument condenser, where it solidifies on the walls. Gradually the porous cake is produced. The pores in the formed product correspond to regions that were occupied by frozen crystals at the beginning of the step.

In the third step, secondary drying occurs. The unfrozen water, which still remains in the cake, is removed by desorption. This step can begin after the sublimation of all ice crystals in the previous step. Usually, a moisture concentration under 1% is required.

It was reported that the ideal freeze-drying medium has certain properties such as a high vapor pressure, a melting point above room temperature, a high viscosity, and so forth. These properties lead to the complete and fast removal of the solvent, providing a more stable cake which is resistant to collapse. A widely-used non-aqueous medium exhibiting the abovementioned characteristics is tert-butanol (TBA) [37–39]. TBA is often used as a co-solvent in combination with water. In this work, TBA was considered to be a suitable co-solvent for two reasons—first, it has properties which make it ideal for use as a freeze-dried medium; second, it completely dissolves hydrophobic fluorescence probes when mixed with aqueous hyaluronan solution. The prepared mixtures can be well homogenized and molecules of hydrophobes equally distributed in the solution around biopolymer chains.

Furthermore, lower molecular weight alcohols (at sufficiently high concentration) are well-known precipitants of hyaluronan from its aqueous solutions and are often used in hyaluronan biotechnological production to isolate it from the raw culture medium. This is a result of dehydrating action of alcohols due to which the hyaluronan hydration shell is disturbed or even removed [40, 41]. These alcohols are also claimed to disrupt the aggregates formed by hydrophobic interactions and ethanol was shown to favor the appearance of hydrophobic domains in chitosan [42].

This pilot study aims at applicability of freeze-drying in solubilizing hydrophobic substances directly with native hyaluronan, evaluate feasibility of this approach and justify (or not) its further development leading up to the drug delivery applications. Three model fluorescence probes were used differing in their hydrophilicity/hydrophobicity and sensitivity to the polarity of the surrounding environment. Two fluorescence techniques, steady-state and time-resolved, were applied.

## Material and methods

### Materials

Native hyaluronan of three different molecular weights (106 kDa, 420 kDa and 1.46 MDa) was obtained from Contipro (Czech Republic). The molecular weights were determined by the producer using the SEC-MALLS method. Stock solutions of hyaluronan were prepared in deionized water to a concentration of 0.5 g∙L^−1^.

Pyrene, prodan, and perylene of fluorescence grade were purchased from Fluka (Czech Republic). Stock solutions of fluorescence probes were prepared in acetone p.a. obtained from LachNer (Czech Republic). The concentrations of stock solutions were 2∙10^−4^ mol∙L^−1^ in the case of pyrene and prodan, and 1∙10^−4^ mol∙L^−1^ in the case of perylene.

Tert-butanol (anhydrous ≥ 99.5%) was obtained from LachNer (Czech Republic).

For the preparation of samples, fluorescence probes in acetone were added to freeze-drying flasks and the acetone was evaporated. Subsequently, 10 ml of TBA was introduced and the samples were mixed on a vibration shaker until probe dissolution. Then, 40 ml of hyaluronan stock solution was added. The samples were stirred constantly until their homogenization. The prepared samples were frozen at −105°C for 12 hours.

Solutions pH was checked and no significant difference between the mixture prepared for freeze-drying and corresponding hyaluronan solution was found; pH was around 5.6.

### Freeze-drying

Frozen samples were transferred onto the drum manifold of a freeze-dryer (VirTis BenchTop 4K ZL). Primary drying was carried out at an ambient temperature of 25°C for 30 hours; the temperature in the condenser was −105°C and the pressure was 10 μbar. Subsequently, secondary drying was performed under the same conditions for the next 18 hours.

After the termination of freeze-drying, the obtained cakes were rehydrated with 20 ml of deionized water and dissolved. Henceforth, the expression “dried sample” will refer to a sample subjected to both freeze-drying and rehydration (i.e. a liquid sample). The final concentration of hyaluronan in the sample was 1 g∙L^−1^ and the concentration of fluorescence probe was 5∙10^−6^ mol∙L^−1^. The whole amount of TBA was assumed to have been removed during the freeze-drying process. The efficiency of the drying process in supporting interactions was monitored using fluorescence spectroscopy. Each sample was compared to its blank solution, which was prepared from fluorescence probe and hyaluronan in water. These blank samples were not subjected to the freeze-drying process.

The residual amount of TBA was determined by a commercial laboratory in dried samples using head-space gas chromatography with FID detection.

### Fluorescence spectroscopy

Prepared samples containing different fluorescence probes and hyaluronan were measured using a Horiba Jobin Yvon Fluorolog at a temperature of 25°C. The structure of the probes is shown in Scheme A in S1 File. They were selected due to their solubility properties in aqueous environment and sensitivity to the polarity of their environment which reflect their structural differences [43]. Pyrene is traditional hydrophobic probe which is still, but sparingly, soluble in water. Pyrene thus fluoresces even from aqueous medium but characteristics of its fluorescence spectra are sensitive to the polarity of its environment. Prodan is soluble both in polar and non-polar environments and its fluorescence maximum is shifted in dependence on the polarity of its environment. Perylene is insoluble in water and does not fluoresce from aqueous environment. Thus it is the “leading probe” for this work.

Samples with pyrene were measured using an excitation wavelength of 335 nm. The emission spectrum was obtained in the range from 360 to 520 nm. During the measurement, the ratio of the first (373 nm) and third (383 nm) vibronic peaks in the scan was monitored. This ratio is called the emission polarity index (EmPI). EmPI indicates the polarity of the microenvironment in which pyrene is soluble. For a polar solvent, the ratio reaches a value of 1.7 and for more hydrophobic solvents, the ratio decreases [31–32].

**Fig 2:**
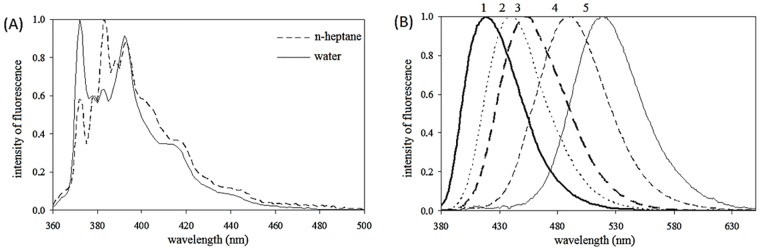
Normalized emission spectra of (A) pyrene in n-heptane and water solution (B) prodan in toluene^1^, chlorophorm^2^, dimethylformamide^3^, ethanol^4^ and water^5^ solution.

Samples containing prodan were excited at a wavelength of 360 nm and the emission spectrum was obtained in the range from 400 to 600 nm. Prodan is a probe that responds to solvent polarity by shifting the wavelength of the maximum peak. Prodan dissolved in water exhibits a maximum peak at 520 nm; for a nonpolar solvent the value is shifted down to 405 nm. If prodan fluoresces from a heterogenic microenvironment of varying polarity, the spectrum is composed of individual peak contributions. The number of peaks corresponds to the number of microenvironments which can be found in the sample [43]. These properties of pyrene and prodan in solutions of different polarity are demonstrated in Fig 2.

The last fluorescence probe used in this study was perylene. In this case, the excitation wavelength was 410 nm and the emission spectrum was collected in the range from 420 to 600 nm. Perylene is a strongly hydrophobic fluorophore, which does not dissolve in polar solutions at all. Therefore, perylene in water provides no fluorescence signal. The polarity of the environment can be monitored by the intensity of fluorescence. From the point of view of the intended future application of the investigated technique in the delivery of hydrophobic drugs perylene was the most relevant model.

### Time-resolved fluorescence spectroscopy

Samples containing perylene and prodan were also characterized using a Horiba Jobin Yvon FluoroCube time-resolved fluorescence spectrometer. The technique was time correlated single photon counting. Measurements were performed at a temperature of 25°C. The basic instrument parameters are given in Table 1.

**Table 1:** Basic instrument parameters for time-resolved fluorescence spectroscopy.

fluorescence probe	excitation wavelength	time-to-amplitude (ns)	repetition rate (MHz)	delay (ns)
prodan	361 nm	50	1	65
perylene	389 nm	100	1	115

All data were analyzed by DAS6 software using models for multiexponential decay with *n* components. The fluorophore could be solubilized within one system in several areas (for example in the water and in the hyaluronan domain). These different environments significantly affect the obtained value of the lifetime of the fluorophore. The relationship of this parameter with the polarity of the environment can be seen in Table 2. The lifetimes of all excited states in our systems were determined using DAS6 software. These values were crucial for assessment of the interactions between the probes and hyaluronan in the studied systems.

**Table 2:** Lifetimes of used fluorescence probes in different solvents and their polarity index (PI).

fluorescence probe	solvent	PI	lifetime (ns)
prodan	cyclohexane	0.2	0.24 [46]
	ethanol	5.3	3.37 [46]
	water	10.2	~ 0.7 (60%) and ~ 2 (40%) [47]
perylene	cyclohexane	0.2	6 [43]
	chloroform	4.1	4.6 [48]
	1,4-dioxan	4.8	4.87 [48]

## Results and discussion

In aqueous solutions, native hyaluronan exhibits a strong hydrophilic character. It has a specific organization in solution, with two competing favorable water structures consistent with maximum degrees of freedom of the ensemble of hyaluronan rotamer and water as a whole [44]. This is the main reason why spontaneous natural interactions with hydrophobic substances are impossible. This was demonstrated by adding pyrene to hyaluronan solution, after which no decrease in EmPI was observed. The hyaluronan chain also contains hydrogen atoms on CH groups forming “hydrophobic patches” [45], which could interact with hydrophobic compounds. However, these areas are inaccessible because of the presence of water molecules. Heatley and Scott showed that hyaluronan can form two stable secondary structures, depending on the water contents in the environment and demonstrating considerable areas of hydrophobic patches [48]. They suppose, for example, that a structure adapted to the water-poor environment of cell membranes is formed and responsible for hyaluronan interactions with membranes or hydrophobic peptide sequences. The main goal in this work was to create the ‘conditions of water shortage’ and thereby make the hydrophobic patches available for interactions. Because hyaluronan degrades at temperatures which are too low to remove hydration water, thermal drying was not possible. Therefore, the method of freeze-drying was utilized in our experimental work to support interactions between hydrophobic fluorescence probes and native hydrophilic hyaluronan in the presence of TBA as a co-solvent.

Fluorescence probes were used to evaluate freeze-drying efficiency and as model hydrophobic substances. Pyrene, prodan and perylene were used as probes and were chosen because of the specific properties of their fluorescence spectra, which can help to identify the polarity of the environment in which the probe is dissolved.

**Fig 3:**
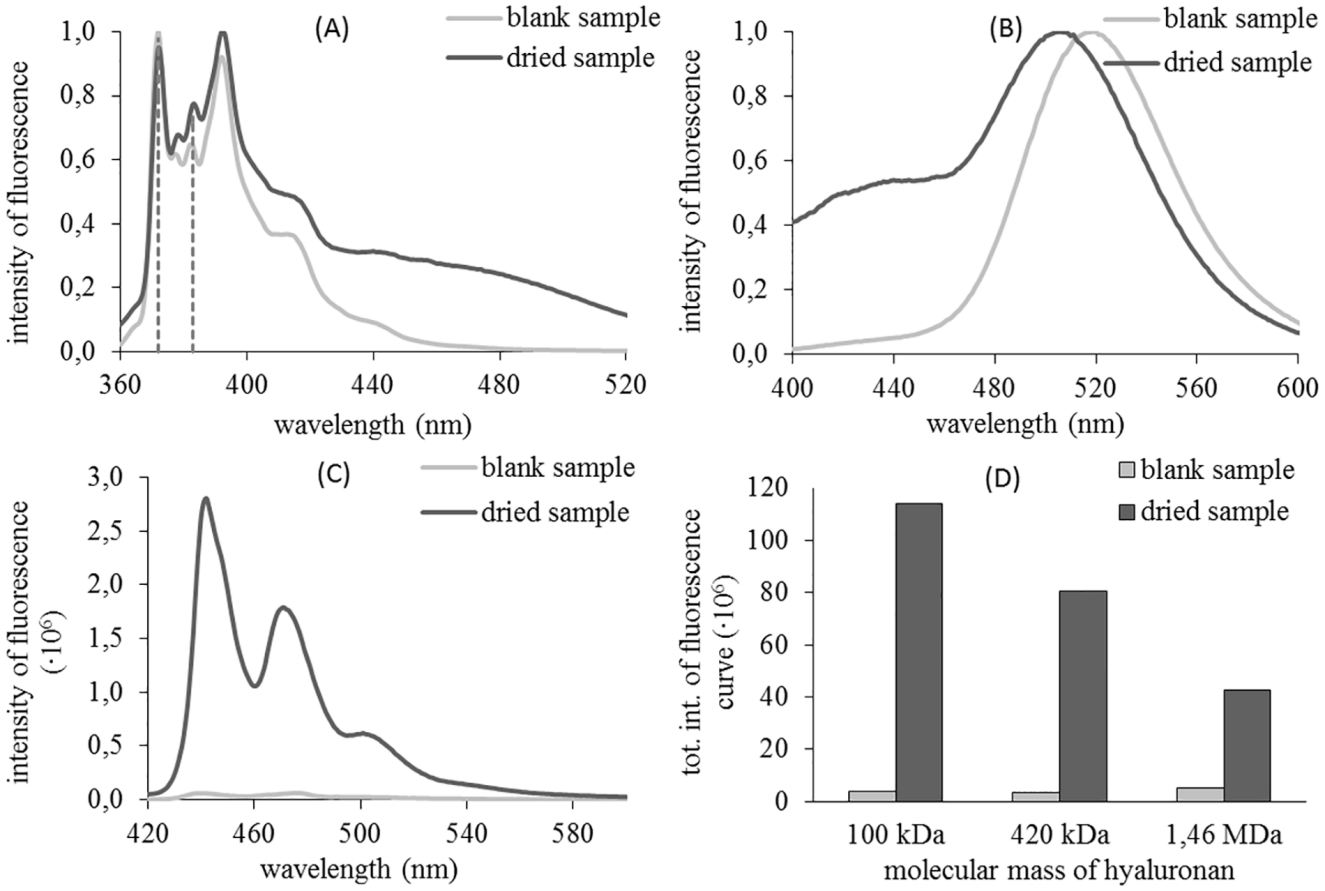
(A) Normalized emission spectra of blank and dried samples with pyrene and HyA 106 kDa, (B) Normalized emission spectra of blank and dried samples with prodan and HyA 106 kDa, (C) Emission spectra of blank and dried samples with perylene and HyA 106 kDa, (D) The decreasing trend of the total integral of fluorescence intensity for dried samples with an increasing molecular mass of hyaluronan and perylene as the fluorescence probe.

The obtained emission spectra for dried and blank samples are shown in Fig 3. For pyrene (Fig 3A), the difference in the intensities of fluorescence for the third vibronic peak is obvious. This significantly affected the value of the determined EmPI. A summary of calculated EmPI for all studied molecular weights of hyaluronan can be found in Table 3. It is clear that the EmPI of blank samples correspond to the polar environment of water solution. Thereafter, we freeze-dried and rehydrated the samples. The determined values of EmPI for dried samples decreased in comparison with samples which did not undergo this treatment. The decrease in EmPI is connected with pyrene emission from more hydrophobic environments. This decrease can be attributed to the pyrene, which is partly bound onto the hydrophobic areas of the hyaluronan chain. The highest molecular weight seems to be less effective. The pyrene fluorescence is composed from signals coming from water and hydrophobic environment. In the case of the highest molecular weight the latter is relatively weaker comparing with the two lower molecular weights. This is attributed to the entrapment of pyrene molecules in aqueous domains of coiled structure of the high molecular weight hyaluronan [8] and preventing them to enter the hydrophobic patches. We will return to this finding below when discussing results obtained with perylene.

**Table 3:** Ratio of the first (373 nm) and third (383 nm) vibronic peaks in the scan for samples with pyrene as a fluorescence probe.

type of hyaluronan	EmPI (blank)	EmPI (dried)
Mw = 106 kDa	1.54	1.28
Mw = 420 kDa	1.53	1.21
Mw = 1.46 MDa	1.53	1.47

In the case of prodan (Fig 3B), the results show that the signals from blank samples were provided only by water. This is obvious from the obtained spectrum, which contains only one peak with a maximum at 519 nm (in the literature [43], 520 nm is reported). Freeze-drying of the samples led to changes in all measured prodan spectra. Subsequently, the emission response had two maxima, and the final curve was the sum of the individual contributions of prodan molecules in environments of different polarity. Due to the summing of the contributions, the “water peak” was shifted to a wavelength around 513 nm. The second peak was at significantly lower wavelengths, as is common in more hydrophobic solvents. Therefore, we suppose that, even in these samples, interactions between this probe and the hyaluronic chain were successfully supported. A summary of obtained results for specific molecular weights of hyaluronan in the samples is shown in Table 4.

**Table 4:** Wavelengths of maximum peaks for samples with prodan as a fluorescence probe and the presence of the second peak in the spectra.

type of hyaluronan	λmax (blank)	second peak	λmax (dried)	second peak
Mw = 106 kDa	519 nm	no	510 nm	yes
Mw = 420 kDa	519 nm	no	515 nm	yes
Mw = 1.46 MDa	519 nm	no	513 nm	yes

The third studied fluorescence probe was perylene (Fig 3C). In this case, fluorescence intensity was the monitored parameter. The fluorescence curve corresponding to the blank sample had almost zero intensity of signal in comparison with the dried sample. Molecules of perylene exhibit a highly hydrophobic character, which is a significant obstacle with respect to perylene’s insolubility in polar environments like aqueous solutions of hyaluronan. Without any “support” of the hydrophobic interactions, the sample with an aqueous solution of hyaluronan was not able to dissolve perylene. This was the main reason for the weak fluorescence signal measured for this sample. After freeze-drying, the signal increased rapidly which is attributed to interactions between perylene and hydrophobic patches on the hyaluronan molecule. Fig 3D displays a comparison of the influence of drying on support interactions in the studied systems for three different molecular weights of hyaluronan. The total integral of the fluorescence curve decreased with the increasing length of the chain. This trend was contrary to the expected results but similar indication was obtained also for pyrene (see above). A longer biopolymer chain should provide more binding sites for a hydrophobic probe. Because the final mass concentration of hyaluronan was the same in all samples, the molar concentrations of the repeating dimeric unit were also equal; however, the hyaluronan molar concentration decreased with increasing chain length. Thus, it seems that the number of chains and not the number of dimeric units play a role. The trend observed in Fig 3D could be ascribed to the presence of longer and more coiled interconnected chains (and their smaller number), inside which further hydrophobic patches still remain screened from interactions.

Interestingly, Brown [8] in her report on entraining small (and much less hydrophobic) onco-chemotherapeutic drugs within the large voluminous domain of hyaluronan found as optimal the tertiary structure of its molecular weight about 800 kDa. Our outputs thus seem to resonate with this finding. When no TBA was present, the resulting spectra of dried samples were close to those of blank samples. Thus, the presence of TBA was essential for the solubilisation of the probes during the preparation of the samples for freeze-drying.

The residual contents of TBA in dried samples ranged between 0.6–1% (vol.) which indicates that the freeze-drying process should be optimized. However, the residual TBA cannot account for the measured fluorescence. For example, the fluorescence intensity of perylene, the most hydrophobic probe, in TBA-water solutions is much lower than that measured for dried samples; intensity measured for dried samples would correspond to aqueous solutions containing at least 12–16% of TBA (cf. Figure A in S1 File).

Time-resolved fluorescence measurements were utilized as the second method for investigating the success of freeze-drying for supporting the interactions in our systems. These experiments were conducted in order to receive signals from microenvironments involving different physico-chemical properties present in the investigated samples. These domains should differ in the lifetimes of the excited states of fluorescence probes. All measured lifetimes are summarized in Table 5 and were compared with known data from literature (Table 2).

**Table 5:** Fluorescence lifetimes (τ) and relative representations for two different fluorescence probes and hyaluronan of different molecular weights (values in italics correspond to scattered light).

	blank		freeze-dried	
	τ (ns)	representation	τ (ns)	representation
prodan				
M_w_ = 106 kDa	0.64	51%	*0*.*01*	*77%*
	1.92	49%	0.95	12%
			2.79	11%
M_w_ = 420 kDa	0.60	55%	*0*.*02*	*74%*
	2.00	45%	1.04	17%
			3.54	9%
M_w_ = 1.46 MDa	0.62	50%	*0*.*02*	*75%*
	1.88	50%	0.87	14%
			2.61	11%
perylene				
M_w_ = 106 kDa			*0*.*05*	*68%*
	no data		3.91	24%
			7.29	8%
M_w_ = 420 kDa			*0*.*05*	*62%*
	no data		3.44	27%
			6.52	11%
M_w_ = 1.46 MDa			*0*.*05*	*68%*
	no data		3.55	20%
			6.99	12%

Prodan is much more soluble in water than perylene. In blank samples, this probe emitted only from water and the combination of lifetimes (about 0.6 ns at 52% and about 2 ns at 68%) corresponded with this fact. Lifetimes obtained from dried samples were shifted to higher values and pointed to more hydrophobic environments. Very short values (marked in italics in Table 5) did not indicate the lifetimes of prodan but signals caused by light scattering; these data are presented to clarify the sum of relative representations.

We did not obtain any data for perylene in blank samples. This probe is strongly hydrophobic and is not soluble in polar solvents. This was obvious from the durations of the measurements. We tried to collect time resolved fluorescence data from blanks but such measurement is based on the arrival of photons at the detector. When a defined amount of photons reaches the detector, the measurement is stopped. In our case, this process took a very long time, because perylene was not soluble in the blank samples and only a few photons in long interval were excited. For dried samples, the situation was completely different. In addition to the values for scattered light, we obtained lifetimes of about 3.5 and 7 ns. These two lifetimes correlate with literature data for hydrophobic solvents.

The lifetimes observed for dried samples were different to those for blank samples. This means the probes were present in different environments of varying polarity. Especially the very hydrophobic perylene probe, as an excellent model of a nonpolar molecule to be solubilized in an aqueous medium, strongly indicated the occurrence of interactions with hydrophobic patches on the hyaluronan chain.

## Conclusions

The results of this pilot work show that the method of freeze-drying can be considered as a technique for supporting interactions between native hydrophilic biopolymer hyaluronan and hydrophobic species. Three fluorescence probes were used to indicate the efficiency of the creation of such interactions—pyrene, prodan and perylene. Each probe exhibits specific behavior dependent on the character of the microenvironment in which it is dissolved. In this work, the most important parameter was the polarity of this microenvironment.

In first part of the study, steady state fluorescence spectrometry was used. Emissions of probes from polar surroundings indicated no interactions between the studied components of the systems. On the other hand, when the fluorescence corresponded to a nonpolar environment, it was assumed that interactions were established and that the method of freeze-drying was successful. The presented results showed that the technique was effective and that hydrophobes were solubilized directly by native hyaluronan. The successful solubilization of hydrophobic probes in the presence of native hyaluronan was also confirmed by time-resolved fluorescence spectroscopy.

This work is a first step in the development of biocompatible and biodegradable carriers for hydrophobic drugs with targeted distribution of the active compound based on native hyaluronan. Though the fluorescence results are promising they should be supported by other techniques. Work on the optimization of the freeze-drying process, on the minimization of residual TBA, and on the characterization of resulting samples (including real drug molecules) is in progress, as well.

## Supporting information

S1 File (DOCX)
