# Supplementary material for: Fluorescence study of freeze-drying as a method for support the interactions between hyaluronan and hydrophobic species
Source: PLoS One. 2017 Sep 8;12(9):e0184558. doi: 10.1371/journal.pone.0184558 (PMC5590968; doi:10.1371/journal.pone.0184558)
Supplement: S1 File — (DOCX) [file pone.0184558.s001.docx]

Supporting information

**Fluorescence study of freeze-drying as a method for supporting the interactions between hyaluronan and hydrophobic species**

Petra Michalicová*, Filip Mravec, Miloslav Pekař

Brno University of Technology, Faculty of Chemistry, Institute of Physical and Applied Chemistry and Materials Research Centre, Purkynova 118, 612 00 Brno, Czech Republic

*xcmichalicovap@fch.vutbr.cz

Scheme A. Fluorescence probes used in this work:

pyrene

prodan

perylene

Figure A. Perylene fluorescence intensity in water-TBA solutions of different composition. The intensity was normalized to the lowest value measured for dried samples under the same conditions.
